# Human circadian phase–response curves for exercise

**DOI:** 10.1113/JP276943

**Published:** 2019-03-18

**Authors:** Shawn D. Youngstedt, Jeffrey A. Elliott, Daniel F. Kripke

**Affiliations:** ^1^ College of Nursing and Health Innovation and College of Health Solutions Arizona State University Phoenix AZ USA; ^2^ Phoenix VA Health Care System Phoenix AZ USA; ^3^ Department of Psychiatry University of California San Diego CA USA; ^4^ Center for Circadian Biology University of California San Diego CA USA

**Keywords:** PRC, ultra‐short sleep wake schedule, circadian time, phase shift, phase advance, phase delay, 6‐sulphatoxymelatonin

## Abstract

**Key points:**

- Exercise elicits circadian phase‐shifting effects, but additional information is needed.
- The phase–response curve describing the magnitude and direction of circadian rhythm phase shifts, depending on the time of the zeigeber (time cue) stimulus, is the most fundamental chronobiological tool for alleviating circadian misalignment and related morbidity.
- Fifty‐one older and 48 young adults followed a circadian rhythms measurement protocol for up to 5.5 days, and performed 1 h of moderate treadmill exercise for 3 consecutive days at one of eight times of the day/night.
- Temporal changes in the phase of 6‐sulphatoxymelatonin (aMT6s) were measured from evening onset, cosine acrophase, morning offset and duration of excretion. Significant phase–response curves were established for aMT6 onset and acrophase with large phase delays from 7:00 pm to 10:00 pm and large phase advances at both 7:00 am and from 1:00 pm to 4:00 pm. Delays or advances would be desired, for example, for adjustment to westward or eastward air travel, respectively.
- Along with known synergism with bright light, the above PRCs with a second phase advance region (afternoon) could support both practical and clinical applications.

**Abstract:**

Although bright light is regarded as the primary circadian zeitgeber, its limitations support exploring alternative zeitgebers. Exercise elicits significant circadian phase‐shifting effects, but fundamental information regarding these effects is needed. The primary aim of the present study was to establish phase–response curves (PRCs) documenting the size and direction of phase shifts in relation to the circadian time of exercise. Aerobically fit older (*n* = 51; 59–75 years) and young adults (*n* = 48; 18–30 years) followed a 90 min laboratory ultrashort sleep–wake cycle (60 min wake/30 min sleep) for up to 5½ days. At the same clock time on three consecutive days, each participant performed 60 min of moderate treadmill exercise (65–75% of heart rate reserve) at one of eight times of day/night. To describe PRCs, phase shifts were measured for the cosine‐fitted acrophase of urinary 6‐sulphatoxymelatonin (aMT6s), as well as for the evening rise, morning decline and change in duration of aMT6s excretion. Significant PRCs were found for aMT6s acrophase, onset and duration, with peak phase advances corresponding to clock times of 7:00 am and from 1:00 pm to 4:00 pm, delays from 7:00 pm to 10:00 pm, and minimal shifts around 4:00 pm and 2:00 am. There were no significant age or sex differences. The amplitudes of the aMT6s onset and acrophase PRCs are comparable to expectations for bright light of equal duration. The phase advance to afternoon exercise and the exercise‐induced PRC for change in aMT6s duration are novel findings. The results support further research exploring additive phase‐shifting effects of bright light and exercise and health benefits.

## Key points

- Exercise elicits circadian phase‐shifting effects, but additional information is needed.
- The phase–response curve describing the magnitude and direction of circadian rhythm phase shifts, depending on the time of the zeigeber (time cue) stimulus, is the most fundamental chronobiological tool for alleviating circadian misalignment and related morbidity.
- Fifty‐one older and 48 young adults followed a circadian rhythms measurement protocol for up to 5.5 days, and performed 1 h of moderate treadmill exercise for 3 consecutive days at one of eight times of the day/night.
- Temporal changes in the phase of 6‐sulphatoxymelatonin (aMT6s) were measured from evening onset, cosine acrophase, morning offset and duration of excretion. Significant phase–response curves were established for aMT6 onset and acrophase with large phase delays from 7:00 pm to 10:00 pm and large phase advances at both 7:00 am and from 1:00 pm to 4:00 pm. Delays or advances would be desired, for example, for adjustment to westward or eastward air travel, respectively.
- Along with known synergism with bright light, the above PRCs with a second phase advance region (afternoon) could support both practical and clinical applications.

## Introduction

Under usual conditions, exposure to light and other zeitgebers (time cues) entrains the circadian system to the earth's 24 h rotation to promote a species‐specific temporal and environmental niche (Johnson *et al*., 2003). However, in modern society, misalignment between the circadian system and environmental schedules is a common condition that is associated with numerous negative health consequences.

For example, ∼20% of the world's work force are shift‐workers who have a higher prevalence of cancer (Lie *et al*., 2011), depressive symptoms (Asaoka *et al*., 2013), cardiovascular disease (Boggild & Knutson, 1999), disturbed sleep and accidents (Folkard *et al*., 2005) compared to full‐time day workers. Moreover, chronic frequent exposure to rapid transmeridian travel has been associated with cognitive deficits (Cho *et al*., 2000) and mood disturbance (Ballard *et al*., 2006), as well as with reduced longevity in animal models (Davidson *et al*., 2006). Likewise, ‘social jet lag’, associated with relatively delayed sleep timing on non‐work days, has been linked to obesity (Roenneberg *et al*., 2012) and cardiometabolic risk (Wong *et al*., 2015). Accumulating evidence also points to associations of circadian disruption with mood disorders (Lyall *et al*., 2018).

The most fundamental chronobiological tool for correcting circadian misalignment, and thereby potentially alleviating related morbidity, is the phase–response curve (PRC), which describes the magnitude and direction of circadian rhythm phase shifts depending on the time of exposure to a zeitgeber (Johnson *et al*., 2003). For example, the human PRC for bright light is characterized by phase delays (shifts later) to light exposure during evening/late night, phase advances (shifts earlier) to early‐morning light, and smaller or negligible responses to light in the middle of the day (Kripke *et al*., 2007; Revell *et al*., 2012; Crowley & Eastman, 2017). Circadian phase shifts are also strongly influenced by the intensity (Boivin *et al*., 1996), duration (Dewan *et al*., 2011) and wavelength of light (Rüger *et al*., 2013).

Although bright light is regarded as the most potent zeitgeber in humans, its phase‐shifting efficacy (e.g., for amelioration of jet lag) has been less than might be predicted by some laboratory experiments (Samel & Wegmann, 1997). Moreover, many blind individuals have limited circadian synchronization to bright light (Klerman *et al*., 2002) and there are conflicting data regarding whether older adults are less responsive to circadian synchronization to light (Duffy *et al*., 2007; Kim *et al*., 2014; Kripke *et al*., 2007). Bright light can also elicit adverse side effects in susceptible individuals (Terman & Terman, 2005). Thus, exploring alternative and/or adjuvant zeitgebers is worthwhile.

An extensive literature has established that exercise can profoundly influence the circadian system in rodents (Bobrzynska & Mrosovsky, 1998; Gannon & Rea, 1995; Marchant *et al*., 1997; Mistlberger *et* al., 1997; Reebs & Mrosovsky, 1989) and that the phase timing and waveform of the exercise PRC in nocturnal animals is approximately the reciprocal of the light PRC (Reebs & Mrosovsky, 1989). In humans, there is also compelling evidence that exercise can elicit significant phase‐shifting effects (Buxton *et al*., 1997, 2003; Edwards *et al*., 2002; Van Reeth *et al*., 1994) and can facilitate re‐entrainment to a shifted light–dark and sleep–wake cycle (Barger *et al*, 2004; Baehr *et al*., 1999; Eastman *et al*., 1995; Miyazaki *et al*., 2001; Yamanaka *et al*., 2014). Moreover, an attractive benefit of enhancing circadian entrainment with exercise is its unique potential to reduce the health risks associated with circadian misalignment (Lewis *et al*., 2018).

However, the extant literature on the influence of exercise on the human circadian system has had many limitations, including inadequate control or measurement of other zeitgebers or other stimuli that can mask circadian rhythm measurement, and testing of insufficient numbers of subjects in a narrow range of times across the 24 h day. These shortcomings have led to a failure to clearly establish the waveform and amplitude of the human exercise PRC.

Moreover, because previous research has been limited mostly to young male subjects, the generalizeability of the findings is unclear. Animal studies have shown relatively reduced phase‐shifting effects of exercise in older animals (Mrosovsky & Biello, 1994) and sex differences in response to other non‐photic zeitgebers (Goel & Lee, 1995).

The present study aimed: (i) to measure exercise PRCs using multiple circadian markers and (ii) to compare exercise PRCs between young *vs*. older adults and women *vs*. men.

## Methods

### Ethical approval

The study was approved by the USCD Office of Human Subjects Protection. The study conformed to the standards set by the *Declaration of Helsinki*, except for registration in a database.

### Participants and recruitment

Participants were 101 healthy, physically active adults, comprising 48 adults aged 18–32 years (26 women and 22 men; 65% White) and 53 adults aged 59–75 years (29 women and 22 men; 91% White) (Table 1). Participants were recruited by word of mouth, flyers and newspaper advertisements for a study that would involve either bright light or exercise. The PRCs for participants who were randomized to bright light have been reported previously (Kripke *et al*., 2007). The present study reports only the exercise data.

**Table 1:** Baseline characteristics of the participants (mean ± SD)

	Young adults			Older adults		
	Women	Men	Mean	Women	Men	Mean
Age (years)	22.5±3.3	24.7±4.0	23.5±3.7	65.8±4.9	66.6±4.5	66.1±4.7^a^
Height (cm)	165.1±5.4	178.0±6.7	171.0±8.8	160.8±6.2	174.0±7.3	166.2±9.3^a^, ^b^
Weight (kg)	63.2±8.5	78.7±9.5	70.2±11.8	62.3±7.3	77.4±10.4	68.5±11.4^b^
Home bedtime	12:06 am ±1.0h	12:44 am ±1.2h	12:23 am ±1.1 h	10:05 pm ±1.1 h	10:24 pm ±1.1 h	10:13 pm ±1.1 h^a^, ^b^
Home wake time	8:04 am ±1.2 h	8:49 am ±1.1 h	8:23 am ±1.2 h	6:23 am ±1.1 h	6:15 am ±1.1 h	6:20 am ±1.1 h^a^
Home sleep duration	400.5±63.5	407.3±45.9	403.1±56.8	412.3±44.2	355.5±62.7	388.6±59.2
CESD	5.3±4.5	4.2±5.2	4.8±4.8	3.9±4.4	3.46±4.3	3.6±4.3
V˙O2 max (mL kg^–1^ min^−1^)	43.8±6.7	49.2±7.0	46.2±7.3	28.8±6.1	35.9±10.4	31.7±8.8^a^, ^b^
aMT6s mesor	744.3±468.4	780.7±614.7	760.9±534.7	393.2±337.4	373.2±321.7	384.9±328.0^a^
aMT6s Acrophase	3:38 am ±1.49 h	4:39 am ±1.85 h	4:05 am ±1.73 h	2:17 am ±1.50 h	3:39 am ±1.53 h	2:51 am ±1.64 h^a^, ^b^
aMT6s onset	10:43 pm ±1.59 h	12:01 am ±1.86 h	11:19 pm ±1.82 h	9:18 pm ±1.72 h	10:50 pm ±1.71 h	9:58 pm ±1.87 h^a^, ^b^
aMT6s offset	8:21 am ±1.58 h	9:02 am ±2.14 h	8:40 am ±1.86 h	7:14 am ±1.78 h	8:31 am ±1.53 h	7:48 am ±1.78 h^a^, ^b^
aMT6s duration (h)	9.6±1.1	9.1±1.4	9.4±1.3	10.0±1.3	9.7±1.2	9.8±1.3

CESD, Center for Epidemiologic Studies‐Depression.

a Significant age difference.

b Significant sex difference.

Initial screening was based on several questionnaires. To help ensure participant safety, inclusion criteria included self‐reported regular exercise ≥3 days per week, for ≥20 min day^–1^, at an intensity of ≥ 60% maximal effort. Exclusion criteria included recent shift‐work experience (previous 2 months) or travel across multiple time zones (previous 4 weeks); abnormal sleep–wake schedule (i.e., reported bedtime before 9:00 pm or after 1:00 am; wake time before 5:00 am or after 9:00 am); poor sleep; depressed mood [Center for Epidemiologic Studies‐Depression Scale (CES‐D) > 16] (Radloff, 1977); use of medications that are likely to distort melatonin excretion or cardiovascular responses to exercise; having more than one major risk factor for coronary artery disease; having any major symptom or sign of cardiopulmonary disease; or any physical or mental health condition that would contraindicate participation in vigorous exercise or other rigors of the experiment.

Prospective participants who appeared to be suitable based on initial screening questionnaires were given a laboratory orientation and further explanation of the protocol. After the orientation, participants provided their written informed consent approved by the USCD Office of Human Subjects Protection.

### Medical screening

Two to 4 weeks before commencing the laboratory protocol, a fasting blood draw was taken to confirm an absence of pathological levels of serum cholesterol, lipoproteins or blood glucose. Final screening at 1–2 weeks before the laboratory protocol consisted of a medical history interview and physical examination, including a physician‐supervised ECG (12‐lead) at rest and during a maximal graded treadmill exercise test (V˙O2 peak ) to volitional exhaustion. These tests further established the absence of cardiovascular disease and confirmed the participants’ capacity to safely perform treadmill exercise. Following any indications of exercise‐induced ischaemia or arrhythmia (*n* = 3 cases), testing was terminated and the participants were referred to a cardiologist.

The attainment of V˙O2 peak  was defined by either a plateau of oxygen consumption with increasing work rate or by heart rate attainment within 10 beats min^–1^ of age‐predicted maximum plus a respiratory exchange ratio greater than 1.10. On‐line metabolic measurements of expired air were made using a Medgraphics Metabolic Cart (MGC Diagnostics Corporation, Saint Paul, MN, USA). Participants who successfully passed all screening were scheduled for home and laboratory observation.

### Home monitoring

During the week prior to laboratory recording, participants maintained stable sleep–wake schedules (i.e., bed and wake times not varying by >90 min), with timing consistent with their usual habits. Adherence to a stable schedule was verified by continuous assessment of activity and illumination with a wrist‐worn actigraph and a daily sleep log. Participants were asked to maintain their usual pattern of exercise, which was monitored with a daily exercise diary.

Participants were also asked to abstain from alcohol and caffeine for 2 days before entrance into the laboratory. Gradual tapering of caffeine consumption was encouraged. Depressed mood was assessed at the end of baseline with the CES‐D (Radloff, 1977).

### Ultrashort sleep–wake cycle

Participants entered the laboratory at 9:30 am on a Monday and remained for 113–134 h (4.7–5.6 days; until Friday night or Saturday morning). Each participant was assigned to a studio‐apartment room, which was maintained at 20–21 °C. At the time of entry into the laboratory, participants were assigned to exercise at one of eight counterbalanced times‐of‐day or night (described below).

**Figure 1:**
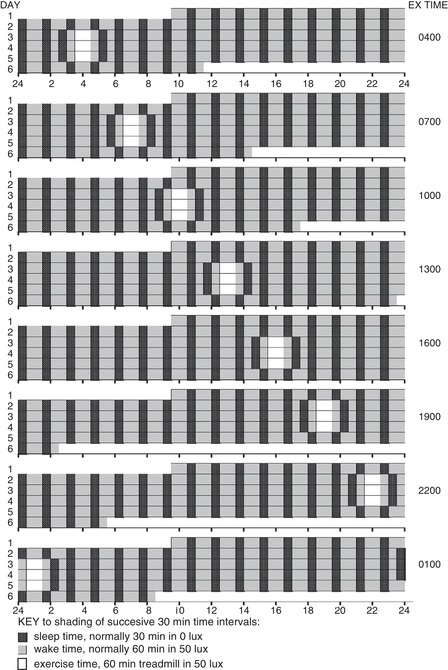
Experimental protocols Each line on the ordinate represents one 24 h day from midnight to midnight (abscissa). Participants arrived in the laboratory at 9:30 am on day 1. An ultrashort sleep–wake cycle, involving 60 min for wake in <50 lux light (grey shaded bars), followed by 30 min for sleep in <0.5 lux (black shaded bars), began immediately and continued for 4.7–5.6 days. Three consecutive treatments (1 h exercise) commenced after 38–54 h of the ultrashort sleep–wake cycle (baseline) at one of eight laboratory clock times (days 2–5, white bars). Circadian phase was assessed during the final 24 h of baseline preceding the first exercise treatment (ending ∼1.5 h before the beginning of the first 1 h exercise) and again for the final 24 h starting 6 h after the third exercise bout (Fig. 2).

After a brief orientation, participants commenced a precise 90 min ultrashort sleep–wake schedule, consisting of a 60 min awake interval, followed by a 30 min interval for sleep, repeated for the duration of the laboratory protocol (Fig. 1). During the 60 min wake intervals, the participants’ rooms and the adjacent hallway were maintained at <50 lux average eye‐level illumination, and participants were required to remain awake and out of bed. Wakefulness was confirmed by actigraphic recording, 24 h video monitoring and direct staff contacts. During each 30 min sleep interval, participants lay down in bed in their darkened (<0.5 lux) sound‐attenuated studio rooms and were requested to try to sleep.

Participants abstained from alcohol and caffeine throughout the laboratory recording and were encouraged to eat small snack‐like meals around the clock. A standardized and constant diet given 16 times a day was not implemented because we considered that this would be too aversive for 5 days, and also because research suggests that minor differences in caloric or food composition across the day/night would have minimal effects on the central circadian pacemaker (Krauchi *et al*., 2002). However, participants were asked to drink at least 200 mL of water or other drink every 90 min to facilitate providing urine samples every 90 min.

Although participants were not isolated from social interaction or from information about the time‐of‐day, they were encouraged to stay in their studio rooms to standardize lighting exposures. Visitors were permitted outside of the 30 min sleep intervals, and participants were able to visit each other or with research staff. Watching video movies (less than 10 lux), working at computer games (less than 8 lux), reading, etc., were permitted *ad libitum*. Exercise was not permitted outside of the experimental treatments (described below).

Through equivalent round‐the‐clock distribution of behavioural and environmental stimuli, the ultrashort sleep–wake cycle unmasks circadian rhythms from these stimuli that can otherwise influence their measurement. In previous studies conducted in our laboratory, under equivalent behavioural and physical conditions including the identical 90 min ultrashort sleep–wake and lighting schedule, we estimated that the urinary 6‐sulphatoxymelatonin (aMT6s) rhythm displayed an endogenous free‐running period of 24.32 h (i.e., on average, aMT6s acrophase delayed ∼19 min day^–1^) (Kripke *et al*., 2005, 2007). In comparison with other circadian rhythm measurement protocols (e.g., constant routine or forced desynchrony), we consider the ultrashort sleep–wake cycle method to be superior for determining PRCs to 3 days of consecutive exercise stimuli.

#### Baseline period of the ultrashort sleep–wake cycle

The duration of the baseline period prior to the first exercise bout varied from 30 h to 53 h (Fig. 1). Commencing the laboratory study at the same time for all participants allowed the research team to inform participants of their treatment and laboratory schedule after their arrival in the laboratory, reducing the likelihood of participants modifying their baseline behaviour in ways that might alter their baseline circadian rhythms.

#### Exercise treatments

During each of the 3 days or nights following baseline, each participant performed exercise for 1 h, centered at one of eight counterbalanced times of day or night: 1:00 am, 4:00 am, 7:00 am, 10:00 am, 1:00 pm, 4:00 pm, 7:00 pm or 10:00 pm (Fig. 1) and at the same clock time across the 3 days.

The ultrashort sleep–wake cycle was maintained during the 3 days and nights of experimental treatments. However, to better accommodate the 1 h exercise stimuli, participants were awake for 2 h associated with each exercise bout: 30 min to prepare for exercise (e.g., stretching), 60 min to exercise and 30 min to shower and cool down. To avoid longer wake intervals, 30 min sleep intervals preceded and followed the 2 h wake periods at times of day/night that were designated for wake during the baseline period.

The exercise consisted of 1 h of treadmill walking or running at 65–75% of heart rate reserve (HRR), which was computed for each participant based on his/her maximal heart rate (HR_max_) obtained during the V˙O2 peak  test, and his/her recumbent resting heart rate (HR_rest_) obtained following awakening from one of the morning sleep intervals during the baseline period: 65% HRR = HR_Rest _+ [(HR_max_ – HR_rest_) × 0.65].

For example, for a HR_max_ and HR_rest_ of 160 and 60 beats min^–1^, respectively, the 65–75% HRR zone was between 105 and 115 beats min^–1^. The intent was to provide a moderately challenging exercise stimulus for these aerobically fit individuals.

This zone of intensity was maintained by varying treadmill speed and/or elevation while monitoring heart rate with a Polar heart rate monitor (Polar Electro Oy, Kempele, Finland), which stored heart rate once per minute and sounded an alarm when the participant's heart rate was outside of the desired zone. A high‐powered electric fan helped cool the participants. To ensure safety, research staff stood beside participants during each exercise bout.

#### Post‐treatment period

**Figure 2:**
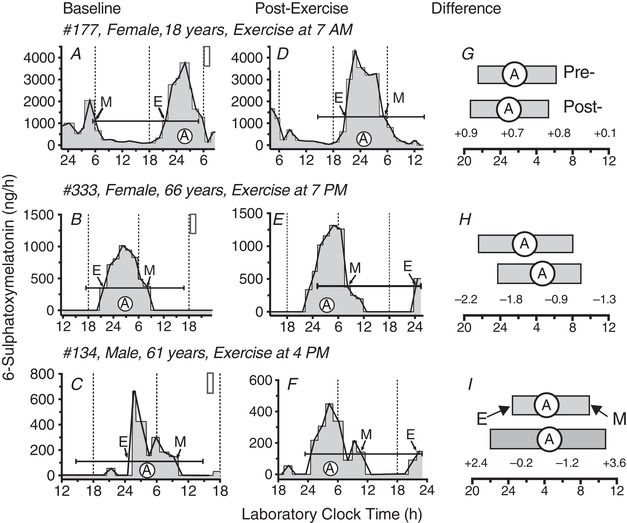
Example aMT6s time series Circadian rhythms of urinary aMT6s excretion (ng h^–1^) are shown for two female participants (177,133), aged 18 years (top row: *A*, *D*, *G*) and 66 years (middle row: *B*, *E*, *H*) and one male participant (134), age 61 years (bottom row: *C*, *F*, *I*). Grey shaded areas (histogram plots in *A*–*F*) represent aMT6s time series used for circadian phase and waveform assessment (baseline *A*–*C*; post‐exercise *D*–*F*). White filled vertical rectangles (*A*–*C*) depict the laboratory clock time of the first of the three daily 1 h exercise bouts (Fig. 1). Cosine curves were fit to the 24 h prior to the first exercise treatment and again to the final 24 h in the laboratory. Capped horizontal lines represent the mesor (cosine fitted mean, ng h^–1^) and 24 h time‐span of pre‐ and post‐treatment cosine fits. White filled circles (A) represent clock times of cosine acrophases (fitted peak times). Thin lines connect temporal midpoints (ng h^–1^) of successive collection intervals. Times of aMT6s onsets (E) and offsets (M) are identified by arrows pointing, respectively, to upward and downward crossings of the associated mesor line. Measured changes in circadian aMT6s rhythm parameters are illustrated under Difference in (*G*), (*H*) and (*I*), where durations of nocturnal aMT6s peaks are represented as horizontal filled rectangles (pre‐ above, post‐treatment below) that span between clock times of E and M in (*A*) to (*F*). The time Difference values for E, A and M, and change in peak duration, respectively, are listed (left to right), beneath the three post‐exercise bars, and in Table 2. All individual Difference values such as these were subsequently transformed to the normalized (corrected) circadian phase‐shift and peak duration changes listed in Table 3 and plotted in the PRCs (Figs 3, 4, 5, 6) by subtracting from each the associated mean Difference of the entire sample (Table 2 and Methods).

Following completion of the third exercise bout, the ultrashort sleep–wake cycle was continued for an additional 30 h (Figs 1 and 2), ending at times varying from 2:30 am to 11:30 pm on day 6. At the conclusion of assessment, participants were permitted to sleep *ad libitum* in the laboratory. Participants were offered a taxi ride home, which was particularly encouraged for participants who finished the study late at night or early in the morning.

### Depressed mood

To help monitor possible adverse effects of the experiment, depressed mood was assessed at the end of days 1 and 5 with the CES‐D (Radloff, 1977). The CES‐D was also assessed on follow‐up day 7 to monitor unanticipated after‐effects of the experiment.

### Circadian collections

To assess baseline and final post‐exercise circadian phase, respectively, urine samples were collected during the 30 h immediately preceding the first, and immediately following the last, exercise bout.

Each time that a participant urinated during these 30 h periods, a sample was taken. With few exceptions, urine samples were collected at least once during every 60 min wake period (i.e., at least every 90 min). The time and volume of each sample was recorded and a portion was frozen (–70 °C) for subsequent assays for circadian rhythms of urinary aMT6s (see below).

### Enzyme‐linked immunosorbent assay of aMT6s

The major metabolite of melatonin, urinary 6‐OH‐melatonin‐sulphate or aMT6s, was measured using Bühlmann 96‐well enzyme‐linked immunosorbent assay (EIA) kits (EK‐M6S) purchased from ALPCO, Ltd (Windham, NH, USA). At the usual dilution of 1:200, the analytical sensitivity of the EIA was 0.35 ng mL^–1^ and the functional least detectable dose was 1.3 ng mL^–1^ for coefficients of variation (CVs) <20%. In our laboratory, control urine samples averaging 4–6 ng mL^–1^ gave intra‐ and inter‐assay CVs of 4% and 7%, respectively. Generally, all samples from an individual participant were run at the same time and on the same 96‐well plate (Kripke *et al*., 2005, 2007; Youngstedt *et al*., 1998). Studies show that aMT6s phase markers are highly correlated with comparable plasma melatonin makers (*r* = 0.7–0.8) with aMT6s phase markers generally occurring ∼60 min after comparable plasma melatonin markers (Deacon and Arendt, 1994).

From the aMT6s concentration, the urine volume and the collection times, the aMT6s excretion rate (ng h^–1^) was computed for each collection interval (the interval between one voiding and the next one) and subsequently associated with each 5 min interval within the collection interval. From this time series of 5 min intervals, the circadian analyses were computed (see below and Fig. 2).

### Circadian metrics

Separate circadian analyses were conducted for the last 24 h of baseline (ending 1 h before the start of the first 1 h exercise bout), and the final 24 h in the laboratory. Data were not used for the first ≥ 6 h after participant entry into the laboratory, nor for the first 6 h after the last exercise bout. These data were excluded to minimize inclusion of acute masking or transient effects on circadian rhythm phase and/or waveform that might occur in response to the initial transition to the laboratory environment or the three exercise bouts (Fig. 2).

Using the least‐squares method available in Action3 software (Ambulatory Monitoring Inc., Ardsley, NY, USA), the best‐fit 24 h cosine function was employed to determine the acrophase (cosine‐fitted time of peak), mesor (fitted mean) and amplitude of the circadian (24 h) rhythms of urinary aMT6s excretion at baseline and following the last exercise bout. To further describe changes in phase and waveform of the aMT6s rhythm, we estimated the circadian timing of the evening rise (onset) and morning decline (offset) of the nocturnal aMT6s peak algebraically from upward and downward crossings of the associated cosine mesor (Fig. 2). Duration of aMT6s excretion was then calculated from the laboratory clock time difference between onset and offset.

Circadian rhythms of oral temperature, urinary cortisol and actigraphic activity were also assessed, but these variables were found to be far less useful as circadian phase markers (Kripke *et al*., 2007) so they are not reported in the present study.

### PRC plotting and analysis

#### Circadian time

The time of the exercise stimulus was expressed as circadian time (CT), calculated for each individual as the clock time of exercise (midpoint of the 1 h bout) minus the difference between that individual's baseline aMT6s phase and the mean baseline aMT6s phase for the entire sample (Fig. 2 and Table 2). This data transformation (normalization) can be represented by: stimulus CT (h) = laboratory clock time of exercise (EX, h) – [(individual baseline phase (h) – mean baseline phase (h)]. For example, for participant #177 (Table 2), exercise was centered at 7:00 am (7.0 h) and baseline aMT6s acrophase was at 1:36 am (1.6 h), whereas the sample mean aMT6s acrophase was 3:26 am (3.44 h). Thus, the CT of her exercise stimulus was at: CT = 7 – (1.6–3.44) = 7 – (–1.84) = 8.84 (i.e., equal to 8:50 am local time for a participant whose baseline aMT6s acrophase was the average of the sample), 1.84 h later than the 7:00 am clock time of EX because her baseline aMT6s onset was earlier than the average of our sample.

**Table 2:** Computation of normalized* circadian responses in aMT6s phase and duration and associated circadian times (CT) of exercise for three representative participants (cf. Fig. 2)

		aMT6s measures in decimal hours			
Subject population/individual traits	Time period/aMT6s variable	Acrophase	Onset	Offset	Duration
Means:	Baseline^1^	3.44	22.62	8.22	9.62
(all eligible data)	Post‐exercise	4.66	23.84	9.22	9.42
	Difference^2^	–1.21	–1.22	–1.00	–0.20
Selected subjects:					
#177, female, age 18 years	Baseline	1.60	21.50	6.10	8.60
EX at 07.00 h	Post‐exercise	0.94	20.60	5.30	8.70
{aMT6s mesor (ng/h):	Difference	+0.66	+0.90	+0.80	+0.10
PRE‐EX = 1093.5	Circadian time^3^	8.84	8.12	–	–
Post‐EX = 1280.9}	Phase shift^4^	+1.88	+2.12	+1.80	+0.30
#333, female, age 66 years	Baseline	2.69	21.60	8.10	10.50
EX at 7 PM	Post‐exercise	4.51	23.80	9.00	9.20
{aMT6s mesor (ng h^–1^):	Difference	–1.82	–2.20	–0.90	–1.30
PRE‐EX = 344.3	Circadian time	17.93	17.82	–	–
Post‐EX = 306.0}	Phase shift	–0.61	–0.98	+0.10	–1.10
#134, male, age 61 years	Baseline	4.29	24.5	9.60	9.10
EX at 16.00 h	Post‐exercise	4.51	22.10	10.80	12.70
{aMT6s mesor (ng h^–1^):	Difference	–0.22	+2.40	–1.20	+3.60
PRE‐EX = 107.7	Circadian time	15.15	14.12	–	–
Post‐EX = 128.2}	Phase shift	+1.00	+3.62	–0.20	+3.80

^*^Normalized circadian phase shifts and duration changes for all eligible aMT6s data are analysed and plotted in relation to CT in PRCs displayed in Figures 3, 4, 5, 6.

^1^Baseline aMT6s acrophase ranged from 12:43 am to 01:48 pm (± SD = 1.79h); baseline aMT6s onset ranged from 6:12 am to 6:00 pm (± SD = 1.96 h).

^2^Difference is defined as baseline (pre‐) minus post‐exercise phase or duration.

^3^CT of exercise is defined as the clock time of exercise (midpoint) minus the difference between that individual's baseline aMT6s phase and the mean baseline aMT6s phase of the entire sample. For example, using the acrophase marker, CT for exercise for participant #177 was 7 – (1.60‐3.44) = 7 – (–1.84) = 8.84.

^4^Phase shift is the normalized phase advance or delay (or duration‐change) of the circadian aMT6s rhythm corrected for the mean drift (delay) across the entire sample.

**Figure 3:**
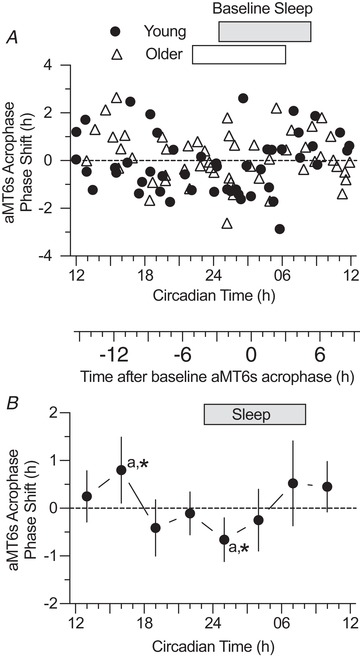
PRC for phase shifts of aMT6s rhythm acrophase (the peak time of the 24 h cosine fit to the ng h^–1^ curve, Figure 2 *A*, phase shifts induced by exercise are shown for 101 participants (closed circles, Young, *n* = 48; open triangles, Older, *n* = 53). Rectangular bars (above graph in Figs 3, 4, 5, 6) represent home‐recorded actigraphic sleep times. The ordinate displays the acrophase shift corrected for the mean phase drift (delay) across the sample. The primary abscissa represents the timing of the mid‐points of the 1 h exercise stimuli transformed (normalized) to CT by adjusting for the difference between each subject's baseline acrophase and the mean baseline acrophase of all participants (Tables 1, 2, 3). *B*, normalized individual phase‐shifts in aMT6s acrophase in (*A*) were averaged into 3 h wide bins of CT stimulus time to yield a PRC curve (mean ± 95% confidence limits) representing all subjects (Young + Older). ANOVA showed a significant time effect (*F*_7,93_ = 2.13, *P* = 0.048). Phase shift means that differed significantly from each other (Tukey's *post hoc* test, *P* < 0.05) are noted with the same lowercase letters. Phase shifts that differed significantly from 0 are indicated with an asterisk. The secondary abscissa placed between (*A*) and (*B*) gives times in relation to aMT6s acrophase at baseline (where 0 is equivalent to CT 3.44).

**Figure 4:**
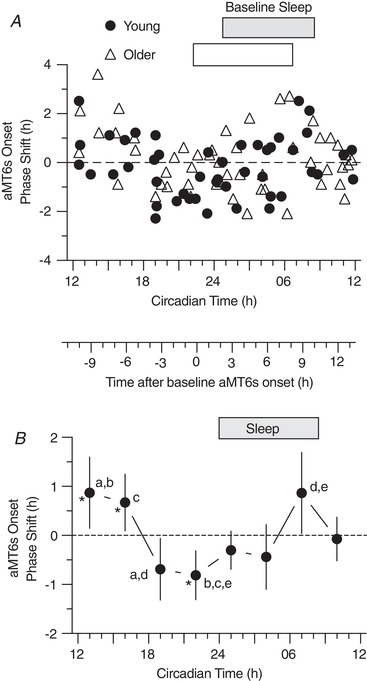
PRC for phase shifts of aMT6s rhythm onset (the evening rise, E in Figure 2) *A*, phase shifts of aMT6s rhythm onsets (ordinate) are plotted for Young (*n* = 48) and Older (*n* = 51) participants with respect to the circadian time (CT) of exercise referenced to baseline aMT6s onset (Fig. 2 and Methods). *B*, the above individual phase‐shifts in aMT6s onset were averaged into non‐overlapping 3 h wide bins of CT to generate a PRC representing all phase shift responses (Young and Older groups combined). ANOVA showed a significant time effect (*F*_7,91_ = 4.92, *P* < 0.001). CT bin mean shifts that differed significantly from one another are designated by shared letters (Tukey's *post hoc* test, *P* < 0.05). Mean shifts differing from zero are designated by asterisks. Both here and in Figs 5 and 6, the secondary abscissa placed between (*A*) and (*B*) gives times of exercise in relation to aMT6s onset at baseline (0 being equivalent to CT 22.62). Other conventions are as in Fig. 3.

**Figure 5:**
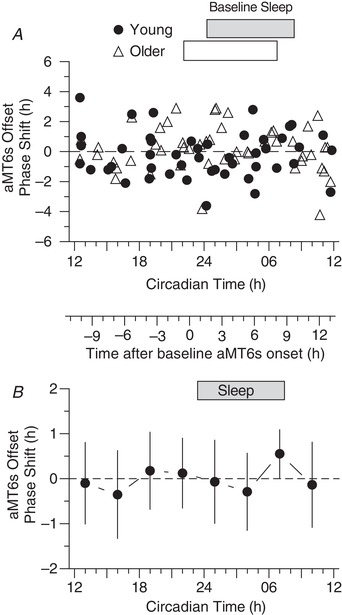
PRC for phase shifts of aMT6s rhythm offset (the morning decline, M in Figure 2) *A*, phase shifts of aMT6s rhythm offsets (ordinate) are plotted for Young (*n* = 48) and Older (*n* = 49) participants with respect to the normalized CT of exercise referenced to baseline amT6s onset (Fig. 2 and Methods). *B*, the above individual phase‐shifts in aMT6s offset were averaged into non‐overlapping 3 h wide bins of stimulus time (CT) to generate a PRC representing all phase shift responses (Young and Older groups combined). Other conventions are as in Fig. 4.

**Figure 6:**
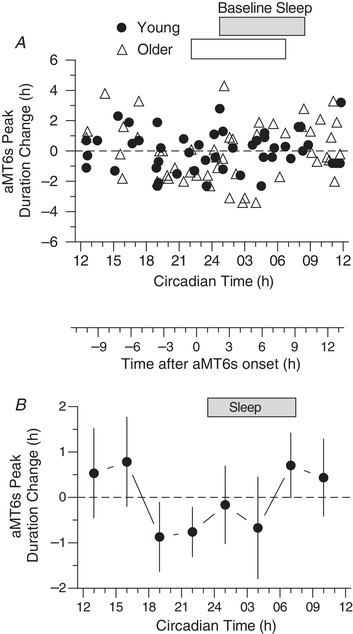
PRC for change in aMT6s rhythm peak duration (i.e., in the width of the nocturnal peak from E to M in Figure 2) *A*, change in aMT6s rhythm peak duration (ordinate) are plotted for Young (*n* = 48) and Older (*n* = 49) participants with respect to the CT of the exercise stimuli (Table 2 and Methods). *B*, the above individual changes in aMT6s duration were averaged into non‐overlapping 3 h wide bins of stimulus time (CT) to generate a PRC representing all responses (Young and Older groups combined). ANOVA showed a significant time effect (*F*_7,89_ = 2.20, *P* = 0.042), whereas *post hoc* tests revealed no time points that differed from one another or from zero. Other conventions are as in Figure 4.

For the PRC for aMT6s acrophase shifts (Fig. 3), baseline aMT6 acrophase was the CT phase marker. For the PRCs for shifts in aMT6s onset and offset, or change in aMT6s peak duration, baseline aMT6s onset was the CT phase marker (Figs 4, 5, 6).

Plotting PRCs with respect to CT permits visualization and statistical analysis of all measured circadian responses to the exercise stimuli on a universal CT scale. This CT scale maps directly to environmental time, where CT 12 is analogous to noon local time and CT 24 (=CT0) is analogous to midnight. CT is adjusted for individual differences in baseline circadian phase to represent a functional best‐estimate of the PRC for the entire sample (Daan *et al*., 2002; Johnson *et.al*. 2003). For example, after correcting for individual differences in baseline phase, CT 2 represents that the stimulus was at a laboratory clock time of 2:00 am for a participant whose baseline phase was equal to the mean sample phase. Figure 2 and Table 2 illustrate the differences measured and the subsequent data transformations for three representative participants.

#### Circadian phase shift response

Changes in aMT6s phase were calculated by subtracting the final phase time (measured on laboratory days 5–6) from the baseline phase time (measured from laboratory days 1–2) (Figs 1 and 2). These individual shifts were then corrected for the mean drift in phase (delay) across all participants. According to convention, phase advances and phase delays are indicated by positive and negative phase shift values, respectively (Figs 3, 4, 5). For change in aMT6s peak duration, positive and negative values indicate increases and decreases, respectively (Fig. 6).

#### Graphing

PRC scatterplots (Figures 3 *A*, 4 *A*, 5 *A*, 6 *A*) graph the magnitude and direction of each normalized circadian rhythm phase shift on the ordinate with CT of exercise on the abscissa. Separate PRCs were derived for aMT6s acrophase, onset, offset and change in peak duration. Figures 3 *B*, 4 *B*, 5 *B*, 6 *B* show plots of the mean ± 95% confidence limits of non‐overlapping 3 h bins of CT.

#### Statistical analysis

Baseline differences in circadian parameters were compared between young and older participants and between women and men with ANOVA. PRCs of each circadian measure were assessed by one‐way ANOVA comparing changes (in phase or duration) among the eight 3 h CT bins, followed by Tukey's *post hoc* comparisons of individual bins. Two‐way CT bin‐by‐age and CT bin‐by‐sex ANOVAS were also conducted.

In addition, whether shifts in the means of 3 h CT bins differed from zero was assessed by ANOVA. ANOVAs and *post hoc* comparisons were also conducted to assess phase shifts relative to the eight laboratory clock times of the exercise treatments.

PRCs can also be evaluated by PRC bisection tests (Kripke *et al*. 2003), which determine whether the pattern of phase advances and phase delays differs significantly from random distribution. However, because the PRC bisection test's assumption of a single phase advance and a single phase delay region was not met, PRC bisection results are not presented.

One participant stopped after 45 min of walking on the first exercise day as a result of ankle soreness. However, he was able to perform the exercise on subsequent days, and so data from this participant were included in the analyses. All other participants were able to perform all of the 60 min exercise bouts, and they all spent more than 50 min h^–1^ within the prescribed zone of intensity (average = 53±3 min).

The aMT6s data from three participants were considered unsuitable for unambiguous measurement of any circadian rhythm parameters as a result of extremely high levels of aMT6s (suspected melatonin intake, *n* = 1) or exceptionally erratic (multiple peaks each day) noisy and/or low amplitude aMT6s variation (*n* = 2). aMT6 onset data were considered outliers for two participants whose values differed >2.5 SD from the mean shift of the associated 3 h CT bin. As a result of multiple peaks, aMT6s offset (and thus, duration) could not be unambiguously identified in two other participants. These exclusions resulted in a sample size of *n* = 101 participants for the aMT6 acrophase PRC, *n* = 99 for the onset PRC and *n* = 97 for the offset and duration PRCs.

## Results

### Participant descriptive statistics

Participant descriptive data are provided in Table 1. Compared with the young participants, the older participants had significantly earlier aMT6 acrophase (*P* < 0.001), onset (*P* < 0.001) and offset (*P* = 0.02), and significantly lower aMT6s mesor (*P* < 0.001). However, the duration of aMT6s excretion did not differ significantly between age groups. Compared with the male participants, the female participants had a significantly earlier aMT6s acrophase (*P* < 0.001), onset (*P* < 0.001) and offset (*P* = 0.008), whereas aMT6s mesor and duration did not differ significantly by sex.

Figure 1 illustrates the experimental protocols including the ultrashort sleep–wake schedule and eight laboratory clock times of the exercise treatments. Example aMT6s (ng h^–1^) time series data are shown in Fig. 2, in addition to the identification of pre‐ and post‐exercise timing of aMT6s acrophase (cosine fitted peak, A), onset (evening rise, E), offset (morning decline, M) and peak duration (time between E and M), pre‐ (baseline) and post‐exercise treatment. The associated individual data are provided in Table 2, which also includes the mean change data (baseline minus post‐exercise difference) that we used to calculate the individual normalized phase‐shift and duration‐change values plotted in Figs 3, 4, 5, 6 and grouped into 3 h wide CT bins for statistical analysis.

### Acrophase‐shift PRCs

PRCs plotting phase‐shifts in aMT6s acrophase are shown in Fig. 3 *A*, which plots individual shifts in which each participant represents one point on the PRC, and Fig. 3 *B*, which plots the mean and 95% confidence limits of non‐overlapping 3 h wide CT bins. ANOVA revealed a significant time effect (*F*_7,93_ = 2.13, *P* = 0.048). Tukey's *post hoc* analyses indicated that the phase delay following exercise at CT 01 (1:00 am) was significantly different than the phase advance at CT 16 (4:00 pm; *P* < 0.05). There were no significant age, sex, age‐by‐time or sex‐by‐time effects.

### Onset‐shift PRCs

PRCs for aMT6s onset shifts are shown in Fig. 4. ANOVA revealed a significant time effect (*F*_7,91_ = 4.92; *P* < 0.001). Tukey's *post hoc* comparisons indicated that the phase advance at CT 13 (1:00 pm) was significantly different than delays at CT 19 (7:00 pm; *P* = 0.023) and CT 22 (10:00 pm; *P* = 0.006); the phase advance at CT 16 (4:00 pm) was significantly different than the delay at CT 22 (10:00 pm; *P* = 0.037); and the phase advance at CT 7 (7:00 am) was significantly different than the delay at CT 19 (7:00 pm; *P* = 0.020) and the delay at CT 22 (10:00 pm; *P* = 0.005). There were no significant age or sex effects, and no age‐by‐time, or sex‐by‐time interactions.

### Offset‐shift PRC

There were no significant time, age, sex, or interaction effects for the PRC for aMT6s offset shifts (Fig. 5).

### Duration‐change PRC

The PRC for changes in aMT6s duration (Fig. 6) showed a timing and waveform similar to that for phase‐shifts in aMT6s onset, as well as a significant time effect (*F*_7,89_ = 2.20, *P* = 0.042), although no significant age, sex or time‐interaction effects were found and Tukey's *post hoc* comparisons revealed no significant differences between individual time points.

### Responses relative to clock time of exercise

Table 3 presents the results relative to the eight laboratory clock times of the exercise bouts. ANOVA showed a significant effect of stimulus time only for phase shifts in aMT6s onset with peak advances at 7:00 am and peak delays from 7:00 pm to 10:00 pm. There were no significant age, sex, age‐by‐time or sex‐by‐time effects.

**Table 3:** Circadian aMT6s rhythm responses^1^ to exercise at 8 times of day (mean ± SD)

Response	ALL^2^	1:00 am^3^	4:00 am	7:00 am	10:00 am	1:00 pm	4:00 pm	7:00 pm	10:00 pm	ANOVA^2^
Acrophase shift:										
Group size	101	13	13	12	13	12	11	15	12	*F* = 1.63
Mean (h)	–1.2	–0.4	0.0	0.4	–0.1	0.7*	0.2	–0.1	–0.6	*P* = 0.137
± SD (h)	1.2	1.0	1.4	1.4	0.9	0.8	1.5	1.0	1.0	
Onset shift:										
Group size	99	13	13	12	13	12	10	15	11	*F* = 3.54
Mean (h)	–1.2	–0.3	–0.1	0.9* ^a,b^	0.1	0.7	0.9	–0.5^a^	–0.7* ^b^	*P* = 0.002
± SD (h)	1.2	1.0	1.4	1.3	0.9	1.1	1.4	1.0	0.9	
Offset shift:										
Group size	97	13	13	12	13	12	10	15	11	*F* = 0.75
Mean (h)	–1.0	–0.4	0.6	0.4	–0.4	0.1	–0.4	0.3	0.1	*P* = 0.634
± SD (h)	1.5	1.9	1.5	1.5	1.5	1.5	1.6	1.3	1.3	
Duration change:										
Group size	97	13	11	12	13	12	10	15	11	*F* = 2.56
Mean (h)	–0.2	0.1	–0.6	0.5	0.5	0.6	1.3^a^	–0.7* ^a^	–0.8*	*P* = 0.019
± SD (h)	1.6	2.1	2.2	1.2	1.4	1.3	1.8	1.2	1.0	

^1^Phase‐shifts and peak duration changes listed under each time (1:00 am to 10:00 pm) were normalized by subtracting from each individual difference (pre‐ minus post‐exercise) the mean uncorrected difference (first column, ALL) calculated from the entire sample (Fig. 2 and Table 2).

^2^Statistics in this column (ALL) are for uncorrected individual Difference values (Table 2) of all eight time points combined. Results of one‐way ANOVA on time of exercise appear in the right‐most column.

^*^Asterisks denote means differing from zero (*P* < 0.05, single sample *t* test). Time point means identified by shared lowercase letters denote same‐row means that differ from each other (Tukey's *post hoc* comparison, *P* < 0.05).

### Depression

Levels of depressed mood (CES‐D) increased significantly from baseline (3.9±4.5) until the last day in the laboratory (7.9±6.4), but they were close to baseline after 1 week of recovery at home (4.9±5.5). No significant interactions of CES‐D with age group or sex were found.

## Discussion

Significant PRCs were observed for phase shifts in the acrophase and onset of aMT6s excretion (Figs 3 and 4), with aMT6s onset appearing to be the more robust of these phase markers. The PRC for change in aMT6s duration (Fig. 6) was also significant, showing a temporal pattern similar to that of the aMT6s onset PRC, whereas the irregular temporal pattern for phase shifts in aMT6s offset was not significant. No significant differences in PRCs were observed between young and older adults or between women and men.

Conflicting results have been reported for other attempts to establish a human exercise PRC (Buxton *et al*., 2003; Edwards *et al*., 2002; Van Reeth *et al*., 1994). One study reported only phase‐delaying effects of exercise performed 5 h before to 4 h after the body temperature nadir (Van Reeth *et* al., 1994). Another study reported phase‐delaying effects from 4 h before to 1 h after the temperature nadir, and phase advancing effects from 3–8 h after the temperature nadir (Edwards *et al*., 2002). A third study found an apparent masking effect or transient advance in melatonin onset assessed 2 h following evening exercise (6:00 pm), which was no longer observed after 24 h (Buxton *et al*., 2003). These studies all involved a small number of young male participants assessed over a small number of time points and/or narrow window of time across the 24 h day.

By contrast, the present study examined phase shifts following exercise in 101 participants, including young and older individuals, men and women, with exercise performed at eight different clock times. Additionally, reflecting the known multi‐oscillator complexity of the human suprachiasmatic nucleus (SCN) clock, the present study examined multiple phase markers selected to differentially track phase shifts in the evening (E) and morning (M) oscillator components of the SCN. Moreover, the potential that measured responses might represent transient or incomplete phase shifts of the multi‐oscillatory SCN clock was intentionally reduced by beginning 24 h circadian phase and waveform assessments at least 6 h after entry in the laboratory and 6 h after completion of the final exercise bout.

The timings of the morning phase advance and evening–night phase delay regions of the exercise PRC are similar to the bright light PRC that we found using the same protocol (Kripke *et al*. 2007). Moreover, it makes intuitive sense that morning and night‐time light and exercise would produce similar clock‐resetting responses to help facilitate circadian entrainment, optimizing daytime functioning and nocturnal quiescence in humans and other diurnal species (Johnson *et al*. 2003).

The early and mid‐afternoon phase advance region was more robust than the morning phase advance, both in terms of its statistical significance and its broader timespan, which included two adjacent time points in the aMT6s onset PRC (CT 13 and 16, equivalent to laboratory exercise at 1:00 pm and 4:00 pm). Evidence of a second phase advance region for afternoon bright light has also been found (Revell *et al*., 2012; Crowley & Eastman, 2017). These results are in contrast to conventional thinking that the circadian system is relatively unresponsive to afternoon zeitgebers, and thus could have numerous practical implications. For example, the many individuals who are unable or unwilling to receive these zeitgebers in the morning could receive phase‐advancing effects of exercise or outdoor light in the afternoon. Conversely, for the goal of delaying the circadian system (e.g., for night shift‐workers), it might be helpful to avoid afternoon exercise or bright light, which advanced the circadian system in the present study, as well as in previous studies (Revell *et al*., 2012; Crowley & Eastman, 2017).

Another novel finding of the present study was the exercise‐induced change in aMT6s duration. The similar waveform for the aMT6 onset shift PRC and the aMT6s duration change PRCs, combined with the flatness of the offset shift PRC, suggest that the duration change PRC was driven mostly by phase shifts in aMT6s onset. To our knowledge, a change in aMT6s duration has not been previously observed in humans in the absence of night‐time light exposure or manipulation of the light/dark schedule to truncate or lengthen the duration of night. Animal studies have clearly established an important role of nightly melatonin peak duration in the photoperiodic regulation of the hypothalamic–neuroendocrine gonadal axis and the seasonality of reproduction (Elliott, 1976; Elliott & Tamarkin, 1994). Although similarly striking neuroendocrine effects have not been found in humans, there is evidence to suggest associations of circadian timing and/or duration of peak melatonin with mood (Meliska *et al*., 2013; Tuunainen *et al*., 2002) and reproductive hormones in humans (Ruhayel *et al*., 2007).

The present finding of similar phase‐shifting effects of exercise in older *vs*. young adults is consistent with the results of Baehr *et al*. (2003). Although not statistically different by ANOVA, the individual PRCs of older adults can be expected to be functionally earlier compared to young adults because three of our studies have shown that baseline aMT6s onset and acrophase were significantly earlier in the older subjects (Kripke *et al*., 2003, 2007) and the present study (Table 1). The lack of sex differences is consistent with the results of Baehr *et al*. (1999).

The amplitude of the aMT6s onset PRC for 1 h exercise was approximately one‐third of that previously reported for participants randomized to 3 h bright light in essentially the same ultrashort sleep–wake schedule (Kripke *et al*., 2007). This amplitude difference could be attributable partly to the difference in duration of these zeitgebers. Similar differences in phase‐shifting effects were observed in a study comparing 1 h *vs*. 3 h of bright light (Dewan *et al*., 2011).

Moreover, studies that have compared similar durations of exercise and bright light have found phase‐shifting responses of similar magnitude. For example, studies by Van Reeth *et al*. (1994) found similar phase‐shifting effects of 3 h of continuous bright light (5000 lux) and intermittent exercise (2.5 h) that was of light intensity (average of 50% of maximal capacity), which was equivalent to moderate walking for most individuals. Likewise, in a within‐subjects design, we found that a 90 min bout of vigorous exercise (65–75% heart rate reserve) elicited an average phase delay that was 84% of the delay elicited by 90 min of bright light (5000 lux) at the same time of night (10:10 pm to 11:40 pm) (Youngstedt *et al*., 2016). Conversely, some studies that led to more negative conclusions regarding the effects of exercise have used protocols that would probably not yield substantially larger effects of bright light on the circadian system (Baehr *et al*., 1999; Edwards *et a*l., 2002).

Nonetheless, far more research is required to establish the extent to which exercise is a practical stimulus for shifting the circadian system. Bright light is probably more convenient than exercise for many individuals, and might be better tolerated for durations of 1 h or more, particularly for older and unhealthy individuals. On the other hand, some individuals would prefer exercise, and there are some circumstances in which exercise could be more practical than bright light, for example, following rapid transmeridian travel (depending on time of day) and military deployments. Moreover, some individuals have adverse reactions to bright light or insufficient sensitivity to its phase‐shifting effects.

In hamsters, phase‐shifting effects of exercise are clearly greater with a longer duration of exercise (Gannon & Rea, 1995) and also with a greater total amount of wheel running in a given time (Bobrzynska & Mrosovsky, 1998), which can be considered as a proxy for exercise intensity. In humans, similar phase shifts were found following exercise of long duration but low intensity and exercise of short duration but high intensity (Buxton *et al*., 1997). Further human dose–response studies manipulating both intensity and duration of exercise will be needed to address the practicality and efficacy of using exercise to shift the circadian system. Although very short bright light pulses have shifted circadian rhythms under extreme laboratory conditions (e.g., continuous bedrest) (Rahman *et al*., 2017), potential circadian phase‐resetting by short duration and high intensity exercise has not been tested.

Perhaps the most promising approach for facilitating circadian entrainment is combining bright light with exercise or other zeitgebers. We found evidence for additive phase‐shifting effects of simultaneous bright light and exercise (Youngstedt *et al*., 2002, 2016). Other studies have revealed additive effects of bright light and melatonin (Burke *et al*., 2013; Revell *et al*., 2006; Crowley & Eastman, 2015). Because the melatonin PRC and the bright light PRC both feature a mid‐afternoon peak in phase advance (Lewy *et al*., 1998; Burgess *et al*., 2010; Revell *et al*., 2012; Crowley & Eastman, 2017), a particularly robust phase advance might be achieved with various combinations of morning bright light and/or exercise and afternoon exercise and/or bright light, and/or melatonin on the same day.

The overwhelming health benefits of exercise suggest that it would also be appropriate for the goal of preventing or ameliorating health consequences of circadian misalignment (Lewis *et al*., 2018). Besides eliciting phase shifts of the central circadian pacemaker, there is accumulating evidence that regular exercise facilitates the synchronization of muscle and other peripheral oscillators (Schroder & Esser, 2013) and enhances the amplitude and temporal stability of actigraphic rest/activity rhythms (Gararulet *et al*., 2017), which have been associated with enhanced survival in various patient samples (Innominato *et al*, 2012).

The strengths of the present study include a large sample size and the use of multiple markers of circadian aMT6s rhythm phase and waveform. Moreover, statistical analysis and graphical presentation were based on the CT of exercise. Additionally, this was the first study to generate complete PRCs describing the clock‐resetting effects of exercise at time points distributed uniformly around the 24 h day, thereby demonstrating peak advances at both CT 7 (7:00 am) and CT 13–16 (from 1:00 pm to 4:00 pm) and peak delays at CT 22 (10:00 pm).

The present study also had limitations. First, in comparison to age‐related norms, the participants were relatively healthy, physically active and aerobically fit. The inclusion criteria were considered important for the safety of the participants, but the results might not generalize to the population. On the other hand, animal studies have demonstrated the most dramatic phase‐shifting effects of wheel‐running exercise in previously inactive animals (Gannon & Rea, 1995). Although these effects might be mediated partly by the novelty of the running wheel, they could be analogous to more robust effects of bright light following dark adaptation (Hébert *et al*., 2002). Thus, conceivably less active/fit individuals could be more responsive to exercise of the same relative intensity/duration or similarly responsive to less vigorous or less prolonged exercise. Future studies should address whether there are differences in phase‐shifting effects in participants with varying levels of fitness and physical activity history.

Another limitation is that the moderately challenging exercise for participants in the present study would have been difficult for less active individuals, and impossible for some individuals. We speculated that higher intensity exercise would produce larger effects based on animal studies indicating greater phase shifts with more intense wheel running (Bobrzynska & Mrosovsky, 1998; Gannon & Rea, 1995). Moreover, evidence indicates that effects of exercise on the circadian system are moderated by serotonergic and neuropeptide Y pathways (Marchant *et al*., 1997), which are activated in an intensity‐dependent manner (Bailey *et al*., 1993; Madsen *et al*., 1993). However, the exercise stimulus of the present study, which involved walking for almost all of the older participants, is achievable with a modest amount of training. Moreover, as discussed above, light intensity exercise has also elicited significant effects on human circadian rhythms (Van Reeth *et al*., 1994).

## Conclusions

In summary, PRCs were established for moderate exercise with no significant sex or age‐group difference in amplitude or waveform. Further dose–response studies in various populations and further studies exploring the temporal dynamics of possible additive effects of using a combination of exercise and light (and/or oral melatonin) stimuli to adjust the phase‐timing of the human circadian system are needed to further understand the efficacy and practical utility of exercise as a therapeutic zeitgeber for the human circadian system.

## Additional information

### Competing interests

The authors declare that they have no competing interests.

### Author contributions

DK, SY and JE designed the study. DK was the PI of the research grant; SY and JE helped write it. SY co‐ordinated the study implementation. JE and SY analysed the results. SY led the writing of the manuscript with the help of JE and DK. JE edited the manuscript. JE prepared all of the figures. All of the authors approved the final version of the manuscript. All authors agree to be accountable for all aspects of the work in ensuring that questions related to the accuracy or integrity of any part of the work are appropriately investigated and resolved. All authors qualify for authorship, and all those who qualify for authorship are listed.

### Funding

Research supported by NHLBI HL61280 and HL095799.
